# Safeguarding Foods and Drugs

**Published:** 1917-01

**Authors:** 


### Abstracts and Selections.

### Safeguarding Foods and Drugs.

In the enforcement of the food and drugs act during the last year, the United States Department of Agriculture officials analyzed 29,833 samples of foods and drugs offered for interstate shipment and for import. A physical examination was made of samples from 76,468 shipments offered for import. Of these foreign shipments, 6353 were found to violate the law in some respects and were either excluded from the country or admitted only after the importers had relabeled them to comply with the law. Of the samples of domestic products analyzed 3535, either because of the nature of the product or because the label on it did not tell the truth, were found to be in violation of the Federal law. In 1364 cases the Department recommended to the Department of Justice that criminal prosecution be instituted against the manufacturers or that the goods be seized. In many cases where there was no evidence of intention to defraud, and where there was merely some easily remedied flaw in the wording of a label, the shippers, after being warned in hearings, voluntarily took steps which made their products fully comply with the requirements. In all, there were held 8715 such hearings, many of which resulted in the prosecutions indicated and the gathering of evidence for a large number of additional cases, which will be forwarded to the Department of Justice.

The Bureau of Chemistry, in its annual report, also calls attention to the fact that through the system of service and regulatory announcements now in use, manufacturers are given due notice of the requirements and thus are enabled voluntarily to make their products conform to the law. In this way the government achieves its purpose, frequently without entering into needless and very expensive litigation.

In the regulatory work, special emphasis has been given to the control of drug products and foods liable to spoilage and pollution. These frequently constitute a serious menace to health. The food inspectors have been instructed to be particularly watchful for interstate shipments of bad eggs, milk, oysters, and spoiled canned goods, and false and fraudulent labeled medicines and spurious, synthetic drugs.

### CURBING FRAUDULENT MEDICINES.

Attempts to counterfeit or adulterate imported drugs have been more common since the recent high price and scarcity of many of these products encouraged their imitation. It is interesting to note that of the 1036 cases terminated in the courts during the year, 198 were brought on account of the false and fraudulent labeling of medicines. In all of these medical cases, save five, the courts found for the government, and this, it is believed, has exercised an important deterrent effect on the vendors of nostrums shipped from one State to another.

The work of controlling the fraudulent labels of medicines and mineral waters has been greatly strengthened by the establishment of a separate office to deal with these matters. At the request of the Secretary of Agriculture an officer of the United States Public Health Service has been detailed to take charge of this work. Moreover, through the close co-operation established with the foods and drugs officials of many of the States, the Department was able to direct the attention of the local authorities to the presence of spurious drugs in their States and, as a result, much of these fraudulent goods in the hands of local dealers and beyond the reach of the Federal authorities were destroyed by State and municipal officers who, in many cases, prosecuted those responsible for the local traffic.

### MILK, EGGS AND OYSTERS.

The co-operation in the sanitary control of the milk supply of small cities described in the report for last year has been extended in Illinois, Iowa, Missouri, Kansas, Nebraska, and in New England. It is proposed to repeat this work year after year, extending it each year to new territory. In some localities bad conditions were found, due in the main to insufficient cooling and careless handling. Perhaps the best results of this work has been that it stimulated some of the local authorities to take up similar work independently so that definite permanent improvement of the milk supply of a number of cities has resulted. The co-operative work on the control of the shipment of decomposed eggs described in the report of last year has been extended to cover much of the territory in which shipments originate so that eggs are now candled before shipment far more than formerly and the spoiled eggs destroyed or fed to poultry and stock. At the same time information given to local officials has helped them to curb local traffic in eggs rejected in candling.

The Bureau of Chemistry, after making co-operative sanitary surveys of oyster beds, issued warnings against the interstate shipment of oysters polluted and doubtful beds and, where these warnings were not regarded, undertook prosecutions. As a result, interstate shipment from such territory was stopped.

### OTHER ADULTERATIONS.

The campaign against the sweating of immature oranges and immature grapefruit so as to give the immature fruit the color of ripe fruit has been successful, largely because of the active help of the greater part of the citrus-fruit producers. Comparatively few sweated, immature oranges were offered during the last year, and it is believed the better quality of fruit resulted in a steadier market so that the producer as well as the consumer benefited.

Other forms of adulteration not already mentioned that received especial attention are the adulteration of scallops and canned tomatoes with water, the substitution of colored starch paste for tomato sauce, the reprocessing of spoiled canned goods, the traffic in cull beans, in decomposed tomato products, in rancid olive oil, in wormy horse beans, the substitution of foreign fat for cacao butter in and the addition of cacao shells to, cacao products, the adulteration of rice bran with rice hulls, the coloring of inferior macaroni and of plain noodles, the misbranding of domestic macaroni in simulation of imported goods, and the adulteration of oats with water or weed seeds.

---

Four per cent of the inhabitants of certain sections of the South have malaria.

